# Human-to-Rat Validation of PMI Biomarkers: A Bidirectional Cross-Species Metabolomics Study Reveals Asymmetric Translation

**DOI:** 10.1021/acs.analchem.6c00202

**Published:** 2026-05-11

**Authors:** Ida Marie Marquart Løber, Liam J. Ward, Albert Elmsjö, Carl Söderberg, Palle Villesen, Kirstine Lykke Nielsen, Henrik Green

**Affiliations:** † Department of Forensic Medicine, 1006 Aarhus University, 8200 Aarhus, Denmark; ‡ Department of Clinical Medicine, Aarhus University, 8200 Aarhus, Denmark; § Bioinformatics Research Centre, Aarhus University, 8000 Aarhus, Denmark; ∥ Department of Forensic Genetics and Forensic Toxicology, National Board of Forensic Medicine, 587 58 Linköping, Sweden; ⊥ Division of Clinical Chemistry and Pharmacology, Department of Biomedical and Clinical Sciences, 4566 Linköping University, 587 58 Linköping, Sweden; # Department of Biomedical and Clinical Sciences, Science for Life Laboratory, Linköping University, 587 58 Linköping, Sweden

## Abstract

Accurately estimating the post-mortem interval (PMI, also known as time since death) remains a major challenge in forensic science due to substantial biological and environmental variability in human cases. While animal models provide controlled conditions for identifying PMI-related metabolic changes, their translational value for human forensics is unclear. This study bridges controlled rat metabolomics with real-world human autopsy samples using LC-MS-based metabolomics and machine learning. We analyzed femoral blood from 435 medico-legal autopsies (PMI 1–10 days) and employed two complementary modeling strategies. An untargeted Lasso model using all 3450 detected features achieved *R* ^2^ = 0.51 and identified 51 consistently selected PMI-informative molecular features. To assess cross-species robustness, we reanalysed rat samples using the same analytical platform. Eighteen of the 51 human-derived features were detected in rats, showing clear PMI-dependent trends and strong correlations with PMI. Two metabolites achieved level 2 annotation and matched known PMI biomarkers from previous animal studies, demonstrating meaningful human-to-rat biochemical overlap. In contrast, a targeted Lasso model based on biomarkers identified in rats performed poorly in humans (*R* ^2^ = 0.10), underscoring limited direct rat-to-human translation. Overall, human-derived biomarkers validated robustly in the controlled rat model proved far more reliable. These findings highlight both the potential and limitations of cross-species PMI biomarker discovery and emphasize the need for improved metadata, especially temperature history, to advance PMI modeling in forensic practice.

## Graphical abstract

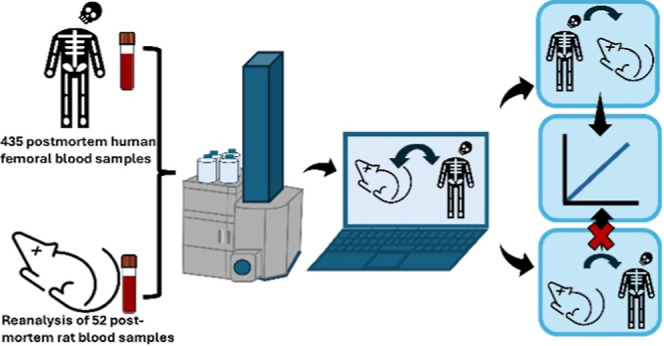

## Introduction

Determining post-mortem interval (PMI) is crucial in medico-legal investigations, providing vital information for reconstructing timelines and understanding the circumstances around unwitnessed deaths and homicides. Despite significant advances in forensic science, accurately estimating the PMI remains a persistent and complex challenge. The biological processes that follow death are highly variable, influenced by environmental conditions and individual-specific factors, which further complicates the PMI estimation process.^1,2^ Traditional methods, such as evaluating rectal body temperature measurements using Henssge’s nomogram and assessing livor and rigor mortis, remain widely used but are inaccurate, thereby limiting their reliability.^1,3^ Furthermore, studies show low consistency among forensic physiologists when estimating PMI from case data, raising concerns about the evidentiary value of such estimates in legal proceedings.^4,5^

Animal models using rodents, sheep, and pigs have commonly been used to investigate post-mortem changes in different tissue matrices, and numerous studies have identified potential biomarkers for estimating PMI.^6–13^ The use of animal models is driven by several key experimental and ethical considerations, particularly the need to study samples with precisely known PMI. However, while these models offer high experimental control, their forensic applicability critically depends on successful translation to human post-mortem biology, which remains a major scientific challenge, particularly in metabolomics. Animal studies benefit from low interindividual variance and controlled conditions, but this very homogeneity may limit their applicability to heterogeneous human populations. Species differences in enzymatic activity, metabolism, and decomposition dynamics further complicate direct translation.

In our previous work, we investigated post-mortem metabolic changes in blood, brain, and muscle using a highly controlled rat model with a PMI range of 0 to 97 h.^12^ Tissue-specific regression models based on a limited set of endogenous metabolites achieved mean prediction errors of approximately 3 h across the entire PMI window and were validated using an independent data set. Importantly, all selected metabolites (except one) were known to be endogenous in humans, providing a rational basis for translational investigation.^12^ Nevertheless, whether these metabolites exhibit comparable post-mortem trajectories and predictive value in humans remains to be systematically tested.

Despite widespread reliance on animal models, systematic animal-to-human translation remains limited. Most studies identify candidate biomarkers in animals without subsequent validation in human post-mortem samples, leaving their forensic relevance uncertain.^7,8,10–12^ Only two prior studies have explicitly attempted cross-species comparison of post-mortem metabolomics findings from animals (rats and sheep) to humans.^6,14^ Both studies demonstrated partial metabolic overlap between species, but predictive models trained on animal data showed reduced accuracy when applied to humans, underscoring the challenge of direct animal-to-human translation. As a result, it remains unclear whether reported PMI-associated metabolites reflect conserved post-mortem processes or species-specific artifacts.

Conversely, human post-mortem metabolomics studies often lack validation in controlled experimental settings, making it difficult to disentangle true PMI-dependent metabolic changes from confounding interindividual variability.^15–17^ This asymmetry between highly controlled animal studies and heterogeneous human data sets represents a central obstacle to translational progress. This lack of bidirectional validation represents a critical gap in the field. Notably, while animal-to-human translation faces inherent challenges due to human heterogeneity, the reverse approach, validating human-derived biomarkers in controlled animal models, offers a potentially more robust strategy, as it tests whether biomarkers identified in complex human data exhibit the expected PMI dependence under controlled conditions.

The present study explicitly addresses the translational gap between animal models and human forensic applications through a novel bidirectional validation framework. Critically, we hypothesize that human-to-rat validation may be more informative than the traditional rat-to-human approach, as human-derived biomarkers can be confirmed under controlled conditions. Using LC-MS-based metabolomics and machine learning, we first identify PMI-associated metabolites directly from human autopsy cases, then validate these in reanalysed rat samples, thereby confirming their PMI dependence under controlled conditions. For comparison, we also test whether biomarkers from our previous rat study translate to human data. By directly interrogating both the strengths and limitations of cross-species translation, and by integrating rat-to-human and human-to-rat translation within a single experimental framework, this study aims to enhance the robustness, interpretability, and forensic utility of metabolomics-based PMI estimation. To our knowledge, this is the first post-mortem metabolomics study specifically designed to achieve systematic bidirectional translation of LC-MS-based findings and machine learning models between animal models and human cases.

## Materials and Methods

### Human Study—Ethics Statement

The human study was approved by the Swedish Ethical Review Authority (Dnr 2019–04530, 2024–05087–02, 2025–02459–02). Due to the retrospective nature of the study, the need for informed consent was waived by the Swedish Ethical Review Authority. All methods were carried out in accordance with relevant guidelines and regulations.

### Human Study Cohort

Autopsy cases admitted to the National Board of Forensic Medicine, Sweden, between January 2021 and December 2024 were considered for inclusion in the study. After the bodies had been found, they were transferred and stored in a chilled environment (5 ± 3 °C) between admission and autopsy (median recorded morgue time from admission at the institute to autopsy: 2 days, minimum: 1 day, maximum: 4 days). Some bodies were additionally transferred and stored in a chilled environment before admission at the National Board of Forensic Medicine due to a shortage of storage space in some departments. Cases were selected from the national boards database of post-mortem cases based on a known date of death (uncertainty up to 24 h, *n* = 7034) and a PMI of 10 days or less. PMI was defined as the time of death until autopsy, when the blood sample was taken. Case matching was based on the group with the least number of cases—those with a PMI of 1 day (*n* = 47). Matching was performed in the following priority order: sex, age and body mass index (BMI). This process resulted in a matched cohort of autopsy cases with PMI groups ranging from 1 to 10 days (*n* = 470). The selected cohort includes individuals aged 18 to 89 years, with BMIs ranging from 11 to 45 (BMI missing for three individuals, later imputed using KNN) and contains autopsy cases from all six regional Departments of Forensic Medicine across Sweden and includes a wide variety of causes of death. Femoral blood samples were collected for toxicological screening and were transported from each regional site to the Department of Forensic Genetics and Forensic Toxicology, Linköping, Sweden. Although sampling is conducted as a multicenter study; sampling, handling, and storage procedures were tightly standardized, with all samples maintained under controlled refrigerated conditions (2–8 °C) and shipped cold overnight. Samples are received and registered the following day, stored under controlled refrigerated conditions, and analyzed on the subsequent day.

### Human Study—Sample Preparation and Sample Analysis

Human post-mortem femoral blood sample preparation and analysis are described elsewhere.^18^ In brief, femoral blood samples were prepared via protein precipitation (acetonitrile/ethanol, 90:10, and 0.075% formic acid) and addition of three internal standards (amphetamine-D8 (retention time: 3.8 min), diazepam-D5 (retention time: 6.78 min), mianserin-D3 (retention time: 9.27 min)). All samples were injected on a UHPLC-ESI-QTOF system (Agilent 1290 Infinity II LC instrument coupled to an Agilent 6545 QTOF with a JetStream interface from Agilent Technologies, Sweden). Separation was performed using a C18 column (Waters AQUITY HSS T3, 150 mm × 2.1 mm, 1.8 μm, Waters Sverige AB, Sweden) using a 12 min gradient elution program. MS data was collected in positive mode. Each analytical run began and ended with a blank drug-free bovine whole blood sample (purchased from a local slaughterhouse) that contained the three internal standards. The samples were analyzed continuously across the four years and not as a combined analytical batch, data were collated retrospectively in accordance with study cohort selection.

### Human Study—Data Processing and Multivariate Analysis

Raw ms1 LC–MS data from the human study were exported to .mzml files (ProteoWizard, version 3.0) and preprocessed using XCMS^19–21^ (version 4.0.2) and CAMERA^22^ (version 1.58) in R (version 4.2). XCMS settings are in supplementary (supplementary page S10). Data was log 1p transformed, and each sample was normalized to the average peak area of the three internal standards specific to that sample. Thirty-five samples were detected as outliers and removed based on one or more of the internal standards deviating more than ±2σ after transformation and normalization. These excluded samples were distributed across the entire PMI period. Thus, 435 cases were included for further analysis and machine learning. We did not observe any clear batch effects, however, we did observe instrumental drift over time, which was corrected using the chosen normalization. Next, all features were used for exploratory data analysis using Principal Component Analysis (PCA) to investigate patterns in the data. Annotated molecules (see the section “Human Study—Annotation”) were retained before any further filtering of the remaining data. Finally, to ensure that each molecule was only represented once, isotopes and adducts were removed from the data; hence, only features of type [M]^+^ and [M + H]^+^ specified by CAMERA were included for further analysis (*n* = 3450). All code is available on GitHub (https://github.com/Idax5975/Human-to-Rat-Validation-of-PMI-Biomarkers). An overview of the study cohort can be found in Table 1.

**Table 1:** Demographic Overview of the Study Cohort^a^

	Cohort values
number of samples	435
females/males	151/284
age	54 (40–65)
BMI^b^	26 (23–30)

a Data are presented as median with quartile range 25–75% in parentheses.

b The BMI of three individuals were imputed using KNN.

### Human Study—Annotation

Annotation was performed on the normalized data before removing isotopes and adducts using an in-house library of reference standards by matching mass and retention times, leading to ID-level 2 and ID-level 3 (*n* total = 122) according to the Metabolomics Standards Initiative.^23^ Features were annotated based on a maximum 5 ppm mass difference and a maximum retention time difference of 15 s. Twenty-four of the annotated biomarkers were reported to be useful for PMI estimation in our animal study.^12^ Additional feature annotation was performed using MassChroViewer (version 1.9.0), leading to ID-level 4 according to the Metabolomics Standards Initiative.^23^ All annotations were checked using the NISTms software (version 2020).

### Machine Learning on Human Cohort Data

Machine learning models were trained and tested in R (v. 4.3.2) using the packages Caret (v. 7.0–1) for Lasso models. The metadata was available for machine learning. Missing BMI values (*n* = 3) were imputed using KNN (R-package VIM, v. 6.2.2) before model training. First, we tried a targeted approach using 10-fold cross-validated (CV) Lasso models using BMI, age, and sex as model inputs, together with the 24 of the 42 biomarkers from the rat study that we could identify in the human data. For each round of cross-validation, we used internal cross-validation to tune the model before it was evaluated on the test fold (machine learning schematic in supplementary, Figure S1). However, as the model in this targeted approach did not perform well, we reevaluated our modeling approach and went for a more untargeted approach. Therefore, we constructed the model once again as described above; however, instead of the 24 annotated biomarkers, we allowed the model to select between the 3450 features in the data set. The significance of the 10-fold CV feature overlap was determined by a conservative permutation test. Code and additional package information are available on GitHub (https://github.com/Idax5975/Human-to-Rat-Validation-of-PMI-Biomarkers).

### Animal Study Resume

The animal study reused for comparison is described in detail elsewhere.^12^ Briefly, 52 rats were euthanised and allowed to decompose up to 4 days at room temperature (19.5 °C–21 °C). Ten rats were sampled each day. Samples (brain, muscle and blood) were extracted and analyzed using untargeted reverse phase UHPLC-qTOF-MS analysis (ACQUITY I-Class UHPLC, Waters, USA, coupled to a Bruker maXis Impact QTOF, Bruker, Germany) using a 19 min gradient method (described in detail elsewhere^12^). Using machine learning, Lasso and Random Forest, a total of 43 biomarkers were selected for modeling. Tissue-specific machine learning regression models, each based on a maximum of 15 biomarkers, showed strong predictive capabilities (*R* ^2^ ranging from 0.970 to 0.990, RMSE ranging from 3.37 h to 5.9 h). Nearly all of the selected annotated biomarkers (42) are endogenous to humans. Knowledge of these annotated biomarkers was used to identify biomarkers of interest in the human cohort data. In the human cohort, we were able to annotate 24 of the 42 rat-selected biomarkers.

### Animal Study Samples Reanalysed at the National Board of Forensic Medicine, Sweden

The extracted samples from the animal study were transported frozen to the National Board of Forensic Medicine, Linköping, Sweden. Here they were thawed and reanalysed using the analytical setup described in the section “Human Study—Sample Preparation and Sample Analysis”. A detailed description can be found elsewhere.^18^ The analysis included an instrument control at the beginning and the end of the analytical run. After injection of the first instrument control sample, the column was conditioned by several pooled QC injections (*n* = 4). The analysis included solvent blank and pooled QC injections for every eighth injection, as well as intrabatch replicates.

### Data Processing of Reanalysed Animal Study Samples

Data from the reanalysis of the rat samples were exported to .mzml files (ProteoWizard, version 3.0) and processed using XCMS^19–21^ (version 4.0.2) and CAMERA^22^ (version 1.58) in R (version 4.3.2). XCMS settings are in supplementary (supplementary page S10). Subsequent correlation analysis was performed using Pearson correlation. Annotations were performed as described in the section “Human Study—Annotation”.

## Results

This study aims to bridge the gap between human cases and animal models, and vice versa, in post-mortem interval (PMI) estimation using LC-MS-based metabolomics and machine learning. In the current study, 470 femoral blood samples from medico-legal autopsies in Sweden (PMI: 1–10 days) were analyzed using UHPLC-ESI-QTOF. After statistics and normalization, 435 samples were included for further analysis. As a comparative reference, a previously published rat study involved 52 rats decomposed at room temperature for up to 4 days.^12^

### Explorative PCA Plot of the Human Cohort

Exploratory data analysis of the human cohort data set using PCA did not reveal a clear PMI signal on the first 10 PC axes (Figure 1). Due to the overall low amount of variance explained, the first 10 PC axes of both raw and normalized data were investigated for any patterns related to the remaining available metadata. However, no clear pattern related to age, sex, or BMI was found (Figures S2, S3 and S4). This suggests that other sources of variation, such as biological variance between individuals, play a dominant role in the data’s signal, which is also previously reported elsewhere.^24^

**Figure 1:**
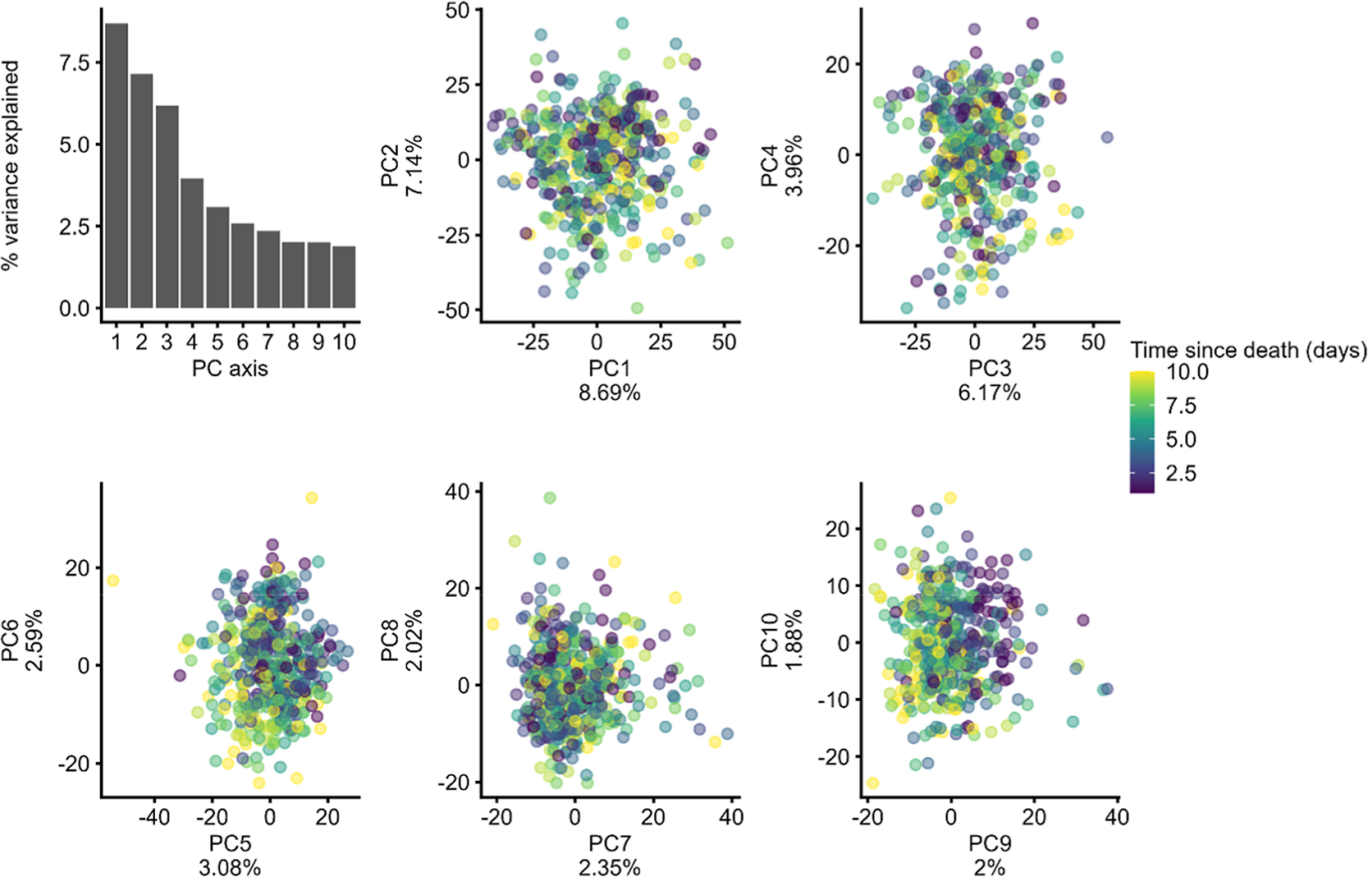
Scree plot and PCA scores-plots of the normalized human data (435 femoral blood samples, 3450 features) showing the first 10 PC-axes colored for PMI.

### PMI Prediction Using Lasso Models

The untargeted Lasso model on the human cohort data achieved an *R* ^2^ of 0.51, demonstrating that a meaningful PMI-related signal exists in the human post-mortem metabolome (Figure 2). Of the 3450 available features, 51 were consistently selected across all 10 cross-validation folds (permutation test, *p* < 0.001), indicating robust PMI-informative markers; all showed stable coefficient signs across folds (21 positive, 30 negative). In contrast, the targeted Lasso model using rat-derived biomarkers on the human cohort showed poor performance (*R* ^2^ = 0.10).

**Figure 2:**
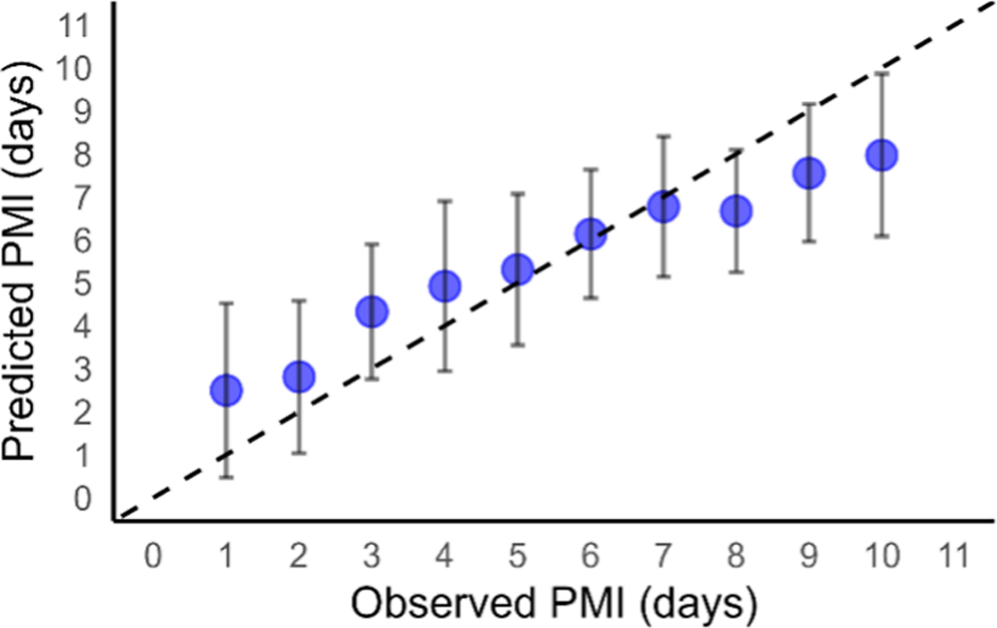
Performance of the untargeted 10-fold cross-validated Lasso model of the human cohort data showing predicted vs observed PMI. Each point corresponds to the mean estimated PMI, while the bars show the standard deviation of each mean.

### Time-dependent Changes of Selected Human Biomarkers

The rat study spanned a PMI from 0–97 h and was conducted at room temperatures (19.5–21 °C), leading to pronounced changes in biomarker concentrations.^12^ In contrast, human corpses were kept at lower temperatures (5 ± 3 °C) for routine storage during most of the post-mortem period. Additionally, PMI of up to 10 days was selected for this study, considering the larger body size of humans compared to rats, and hence the potentially slower cooling of the body. Since the rat study covered a shorter PMI range (0–97 h), direct comparisons beyond 97 h are limited.

Our untargeted Lasso model approach on the human cohort data included a model-driven feature selection, which identified 51 molecular features as the most informative for PMI estimation, as they were selected in all 10 cross-validation folds (overview in Table S1). The distribution of the selected features spanned a broad range of retention times and *m*/*z* values, suggesting contributions from diverse metabolite classes. All 51 molecular features showed consistent time-dependent tendencies but large interindividual variation across the full PMI range (a selection is shown in Figure 3). These temporal trends in selected biomarkers provide valuable insight into post-mortem biochemical processes and may improve the interpretability of future PMI prediction models.

**Figure 3:**
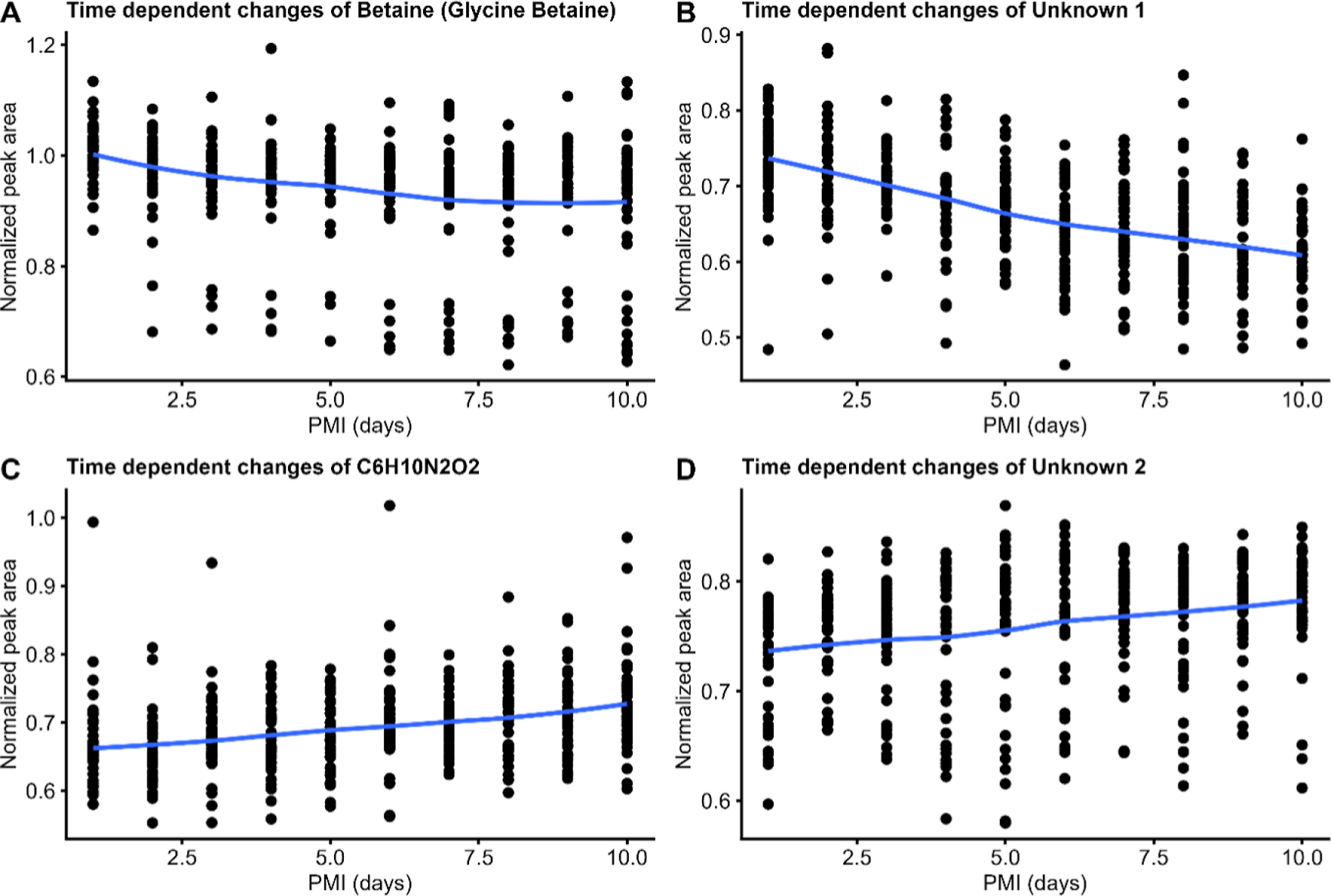
Relative time-dependent changes of a few representative biomarkers from the human cohort data selected by the Lasso model. (A) Relative time-dependent changes of betaine. (B) Relative time-dependent changes of unknown 1. (C) Relative time-dependent changes of C_6_H_10_N_2_O_2_. (D) Relative time-dependent changes of unknown 2.

### Validation of Human Biomarkers in Rat Data

Of the 51 consistent molecular features from the human cohort data, 18 were successfully matched in the data from the reanalysis of rat samples at the National Board of Forensic Medicine in Linköping, Sweden. In the reanalysed rat data set, these 18 matched biomarkers displayed a clear PMI-related separation in the PCA plot (Figure 4A) and showed strong correlations with PMI (Figure 4B–C). Of these 18 features, two were assigned a Level 2 identification based on referencing mass and retention time to an in-house database (Table S1, Figure S5). Fifteen could be assigned a molecular formula, and one remains unknown. Notably, among the annotated metabolites, betaine decreased with increasing time-since-death, consistent with previous observations,^12^ supporting its utility as a cross-species PMI biomarker. Additional details on the annotated metabolites are provided in Table S1.

**Figure 4:**
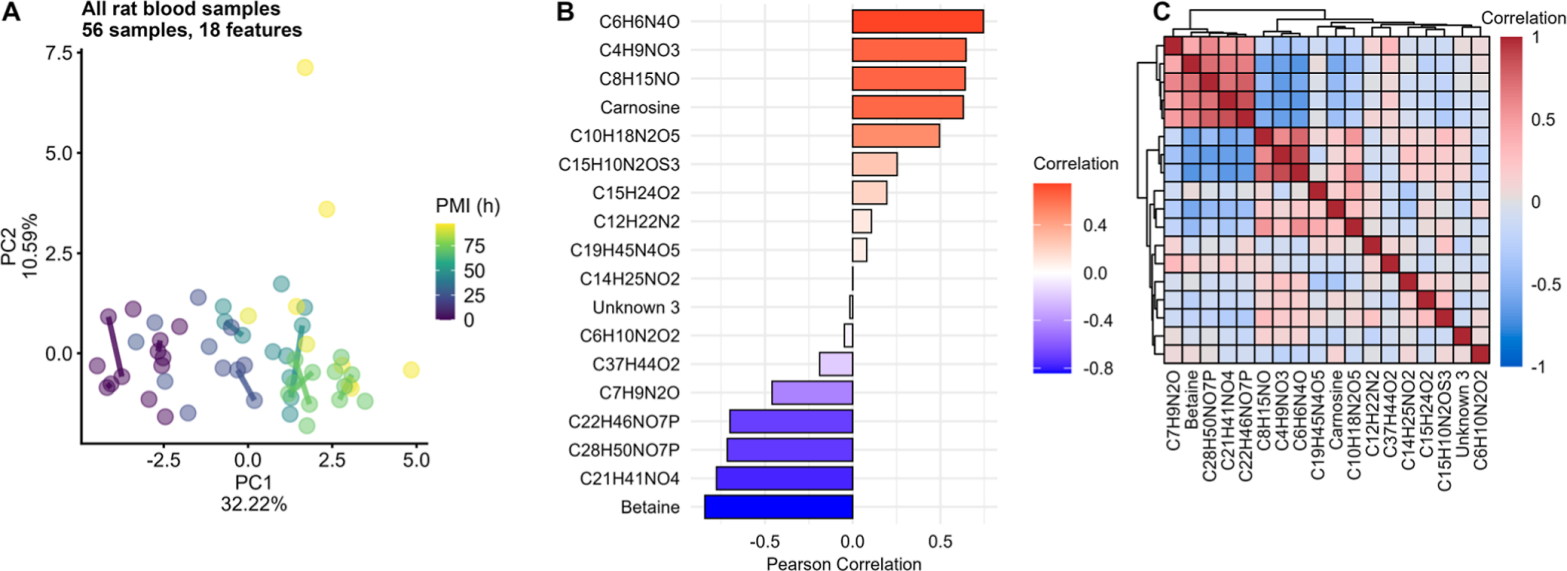
Post-mortem interval (PMI) signal in the reanalysed rat data set using 18 of the 51 biomarkers highlighted in the human cohort study. (A) Principal component analysis (PCA) based solely on the 18 biomarkers demonstrates a clear PMI-related trajectory in the reanalysed rat data. Lines connecting data points indicate technical replicates (samples analyzed multiple times). (B) Individual correlations between each of the 18 biomarkers and PMI. (C) Correlation heatmap of the 18 biomarkers, illustrating their interrelationships and shared PMI-associated patterns.

## Discussion

Animal models have long been instrumental in advancing our understanding of post-mortem biochemical processes and in developing controlled frameworks for PMI estimation. In this study, we set out to rigorously test the translational validity of such models by applying LC-MS-based metabolomics and machine learning across species. Specifically, we evaluated whether PMI biomarkers and predictive models derived from deceased humans could be validated in animal data, and conversely, whether a highly controlled rat model could be transferred to human forensic casework. The results demonstrate a clear asymmetry: translating human-derived PMI biomarkers to rat data was markedly more successful (predictive performance, *R* ^2^ = 0.51) than from rats to humans (predictive performance, *R* ^2^ = 0.10), providing important insight into how animal models may best be used in forensic metabolomics.

The exploratory data analysis using PCA showed no clear PMI signal in the first 10 PC axes of the human data, potentially due to the numerous factors influencing the post-mortem metabolome and instrumental performance variations over time. In contrast, the rat study showed a strong PMI signal with >80% variance explained, which probably reflects highly controlled conditions and low interindividual variance of the animals.^12^ Since PMI accounted for approximately 80% of the variation observed in the rat study, most of the features identified in that context are likely useful for estimating PMI within that specific setting. However, although the rat-selected biomarkers are common endogenous compounds in humans as well, this does not necessarily imply their effectiveness for PMI estimation in the more complex human context, which involves greater interindividual variability and additional confounding factors.

Our primary analytical approach employed an untargeted Lasso model trained on the full human data set (3450 features), which identified 51 molecular features consistently retained across all cross-validation folds (*p* < 0.001). This model achieved an R^2^ of 0.51, demonstrating that meaningful PMI-related structure can be captured despite the complexity of the human post-mortem metabolome. Strikingly, when these 51 human-derived features were interrogated in the reanalysed rat data set, 18 could be matched and exhibited strong PMI-dependent trends. PCA and correlation analyses demonstrated a clear temporal signal in the rat data for these features, providing compelling evidence that human-derived PMI biomarkers capture conserved post-mortem biochemical processes that become more apparent under controlled conditions. Two of these metabolites, betaine and carnosine, achieved level 2 identification, with betaine previously reported as a PMI marker in multiple animal studies across tissue matrices (AH, VH and blood).^7,12^ This convergence supports the notion that animal models are particularly valuable as validation platforms for human-discovered biomarkers, rather than as primary discovery tools for direct human application.

For comparison, we also tested a targeted modeling approach in the human data set using 24 biomarkers previously identified in our rat study. The annotation of only 24 of the 42 rat-selected biomarkers may be due to differences in sample extraction and analytical platform approaches between the rat study and the human cohort. This model performed poorly (*R* ^2^ = 0.10), confirming limited direct rat-to-human translatability. This result highlights several critical limitations of direct animal-to-human translation. Although the selected biomarkers are endogenous to both species, shared biochemical identity does not guarantee shared post-mortem kinetics or predictive relevance. In humans, PMI-related changes in these metabolites are likely obscured by larger biological variability and by metabolites that are more responsive to human-specific post-mortem processes. Moreover, the rat-derived biomarkers were selected under conditions in which PMI dominated variance, a context fundamentally different from heterogeneous human data sets. Collectively, these findings suggest that rat-optimized biomarker panels are insufficient to capture the PMI signal in humans without substantial expansion and investigation, and that animal-derived models should not be assumed to be directly transferable to forensic casework.

Several factors likely explain the observed asymmetry in cross-species translation. The rat study was conducted at room temperature (19.5–21 °C), leading to rapid and pronounced post-mortem metabolic changes over a short PMI window (0–97 h).^12^ In contrast, human bodies were typically stored at 5 ± 3 °C for most of the post-mortem interval, substantially slowing biochemical processes and reducing the early temporal signal. Therefore, we selected a post-mortem interval (PMI) of up to 10 days, as biomarker changes during the first 5 days were limited. Additionally, differences in body mass and cooling rates between species further exacerbate these effects.

Second, incomplete metadata represents a major limitation in human forensic data sets. Preadmission cooling, variable storage histories, and undocumented environmental exposure introduce uncertainty that cannot be adequately modeled as degree-hours or degree-days. The recorded morgue time (median 2 days at 5 ± 3 °C) represents only a limited contribution to post-mortem biochemical progression compared with that observed in the rat study. When expressed as accumulated degree-days (ADD), 4 days of decomposition at ∼20 °C in rats correspond to approximately 80 ADD, whereas the recorded median of 2 days of human storage at e.g. 5 °C accounts for only ∼10 ADD. Even in cases with longer storage (4 days), ADD values remain lower than in the rat experiment. Importantly, however, the total duration and temperature of cooled storage prior to admission are unknown for many of the included individuals, and the bodies have also been exposed to variable ambient temperatures at the scene of death and during transport. These undocumented conditions introduce additional uncertainty regarding the effective thermal history of each case. In addition, minor in vitro changes may occur after sample collection, when the sample is shipped under cooled conditions to the National Board of Forensic Medicine, Linköping, Sweden, for analysis. Nevertheless, the available data clearly demonstrate that refrigeration markedly slows metabolomic changes in human forensic cases, thereby reducing the early PMI signal and contributing to the observed asymmetry in cross-species temporal trajectories. These unknowns, combined with the uncertainty about the time of death, which introduces temporal noise, substantially impair machine learning performance and limit the interpretability of PMI-associated metabolic patterns. Improved environmental, temperature and storage recordings would enable the incorporation of temperature proxies or environmental models into the PMI estimation models, which could further improve the accuracy of the estimated PMI.

Third, the cause of death likely contributes to additional variability.^25–28^ While minimal effects were observed between euthanasia methods in the rat study,^12^ human deaths encompass a wide range of pathological processes known to influence the post-mortem metabolome. This diversity further dilutes PMI-specific signals and complicates direct translation from controlled animal models. Moreover, instrumental drift, which were corrected using normalization to internal standards, could affect data analysis, as the human data set spans a four-year collection and analysis period. Nevertheless, prior studies support the use of retrospective routine data derived from robust, accurate methods with rigorous quality assurance, such as accredited routine procedures, for metabolomics research.^29^ Finally, differences in cohort size and sampling structure likely affected comparability across species. The rat study employed a strictly balanced design with 10 male individuals per PMI time point, providing uniform statistical power across the entire temporal series. In contrast, the human data set contained 47 individuals per day. The fixed cohort size of 47 cases was used to ensure consistent case matching over the full 10 day period. While this approach optimized comparability within the human cohort, thereby eliminating some contributors to heterogeneity, it also introduced asymmetry relative to the controlled sampling strategy used in rats.

Taken together, these findings demonstrate that animal models reveal only fragments of the much more complex human post-mortem metabolic landscape. While rat-to-human translation of PMI biomarkers is limited, the reverse approach, validating human-derived biomarkers in animal models, emerges as a powerful and underutilized strategy. This insight has important implications for future study design: animal models may be best employed not for direct biomarker discovery intended for human application, but rather as controlled systems to confirm PMI dependence and mechanistic relevance of biomarkers identified in human forensic material. To improve PMI prediction models based on human autopsy data, enhanced documentation of post-mortem handling is essential, particularly regarding body storage conditions before and after admission. Ideally, biological samples should be collected as early as possible after body discovery to minimize environmental confounding. Higher temporal resolution of PMI estimates would further strengthen modeling efforts.

This study provides strong evidence that human-derived PMI biomarkers can be robustly validated in animal models, offering a pragmatic and biologically grounded path forward for forensic metabolomics. This bidirectional perspective reframes the role of animal models in PMI research and provides a clearer roadmap for developing reliable, human-relevant molecular tools for forensic practice.

## Conclusions

This study demonstrates that meaningful PMI estimation from human post-mortem blood is achievable using untargeted metabolomics and machine learning. An untargeted Lasso model identified 51 molecular features consistently associated with PMI (*R* ^2^ = 0.51), and crucially, 18 of these human-derived biomarkers exhibited clear PMI-dependent patterns when validated in controlled rat samples, including two with level 2 identification. This human-to-rat validation confirms that the identified biomarkers reflect conserved post-mortem biochemical processes. In contrast, a targeted approach using rat-derived biomarkers performed poorly in humans (*R* ^2^ = 0.10), highlighting the limitations of direct animal-to-human translation. The limited metadata available for human cases, particularly regarding storage conditions and preadmission cooling, remains a key barrier to improving predictive performance in human models. Advancing PMI modeling in forensic practice will require more precise documentation of environmental and case-specific variables, higher temporal resolution of PMI estimates, and ideally, the collection of biological samples at the discovery site. Despite the challenges, this study provides a critical step toward integrating human and animal post-mortem metabolomics and highlights a path forward for refining cross-species PMI biomarkers.

## Supplementary Material



## Notes

Additional tables providing an overview of biomarkers. Additional figures of a schematic machine-learning overview, PCA plots, and curves of biomarker time-dependent changes (PDF)
