# Supplementary material for: Human-to-Rat Validation of PMI Biomarkers: A Bidirectional Cross-Species Metabolomics Study Reveals Asymmetric Translation
Source: Anal Chem. 2026 May 11;98(20):14883–90. doi: 10.1021/acs.analchem.6c00202 (PMC13393081; doi:10.1021/acs.analchem.6c00202)
Supplement: Supplementary file 1 [file ac6c00202_si_001.pdf]

Supplementary Information:

Human-to-Rat Validation of PMI Biomarkers: A Bidirectional Cross-Species Metabolomics Study Reveals Asymmetric Translation

Ida Marie Marquart Løber [1,2,3], Liam J. Ward[4,5], Albert Elmsjö[4], Carl Söderberg[4], Palle Villesen[2,3], Kirstine Lykke Nielsen[1], Henrik Green[4,5]*

[1]Department of Forensic Medicine, Aarhus University, 8200 Aarhus, Denmark; imml@forens.au.dk; klyn@forens.au.dk

[2]Department of Clinical Medicine, Aarhus University, 8200 Aarhus, Denmark

[3]Bioinformatics Research Centre, Aarhus University, 8000 Aarhus, Denmark; palle@birc.au.dk

[4]Department of Forensic Genetics and Forensic Toxicology, National Board of Forensic Medicine, 587 58 Linköping, Sweden; liam.ward@rmv.se; albert.elmsjo@rmv.se; carl.soderberg@rmv.se; henrik.green@liu.se

[5]Division of Clinical Chemistry and Pharmacology, Department of Biomedical and Clinical Sciences, Linköping University, 587 58 Linköping, Sweden

*Correspondence: henrik.green@liu.se

Table of Contents

Table S1: Metabolite overview S3
Figure S1: Machine Learning Schematic S5
Figure S2: PCA scores plot of normalised human data, coloured for age S6
Figure S3: PCA scores plot of normalised human data, coloured for sex S7
Figure S4: PCA scores plot of normalised human data, coloured for body mass index (BMI) S8
Figure S5: Relative time-dependent changes of biomarkers in reanalysed rat samples S9
XCMS settings S10

Table S1: Metabolite overview

Annotation	m/z (ESI+)	ppm difference	Retention time human cohort data (sec)	ID-Level	Re-found in rat data	Retention time rat data (sec)	Annotation from in-house database and MSChroViewer verified by NIST
Betaine (Beta-ine Glycine)	118.0864	-0.76	47	2	X	48	X
Carnosine	227.1147	-3.5	44	2	X	45	X
$C_4H_9NO_3$	120.0654	1.13	54	4	X	46	X
$C_7H_9N_2O$	137.0712	1.9	50	4	X	53	X
$C_8H_{15}NO$	142.1226	-0.29	125	4	X	136	X
$C_6H_{10}N_2O_2$	143.0816	0.67	80	4	X	87	X
$C_6H_6N_4O$	151.0614	-0.25	77	4	X	76	X
$C_{12}H_{22}N_2$	195.1856	0.13	374	4	X	385	X
$C_{15}H_{24}O_2$	237.1849	-0.03	572	4	X	582	X
$C_{14}H_{25}NO_2$	240.1956	-3.13	452	4	X	463	
$C_{10}H_{18}N_2O_5$	247.1293	-0.39	145	4	X	132	X
$C_{15}H_{10}N_2OS_3$	331.0032	1.2	592	4	X	581	
$C_{21}H_{41}NO_4$	372.3111	0.71	607	4	X	612	X
$C_{19}H_{45}N_4O_5$	410.3449	-3.34	606	4	X	603	
$C_{22}H_{46}NO_7P$	468.3088	0.71	592	4	X	598	X
$C_{37}H_{44}O_2$	521.3410	-0.78	635	4	X	630	
$C_{28}H_{50}NO_7P$	544.3403	0.98	611	4	X	619	X
Unknown_1	314.2330		474	5			
Unknown_2	273.0964		59	5			
Unknown_3	131.0898		59	5	X	66	
Unknown_4	136.0754		60	5			
Unknown_5	136.0750		76	5			
Unknown_6	143.0327		33	5			

Unknown_7	175.0342		157	5			
Unknown_8	188.0999		211	5			
Unknown_9	200.0739		161	5			
Unknown_10	204.1529		134	5			
Unknown_11	211.142		135	5			
Unknown_12	219.1948		612	5			
Unknown_13	220.9344		35	5			
Unknown_14	230.1388		147	5			
Unknown_15	241.1839		96	5			
Unknown_16	258.17		251	5			
Unknown_17	268.84		43	5			
Unknown_18	312.2177		424	5			
Unknown_19	330.2274		421	5			
Unknown_20	365.2325		383	5			
Unknown_21	384.3109		607	5			
Unknown_22	446.3268		610	5			
Unknown_23	493.2931		618	5			
Unknown_24	533.3258		432	5			
Unknown_25	591.3179		406	5			
Unknown_26	600.4688		619	5			
Unknown_27	714.5090		642	5			
Unknown_28	821.6188		638	5			
Unknown_29	822.5507		639	5			
Unknown_30	837.7414		653	5			
Unknown_31	844.6076		640	5			
Unknown_32	909.5562		654	5			
Unknown_33	936.7519		654	5			

Machine Learning Schematic

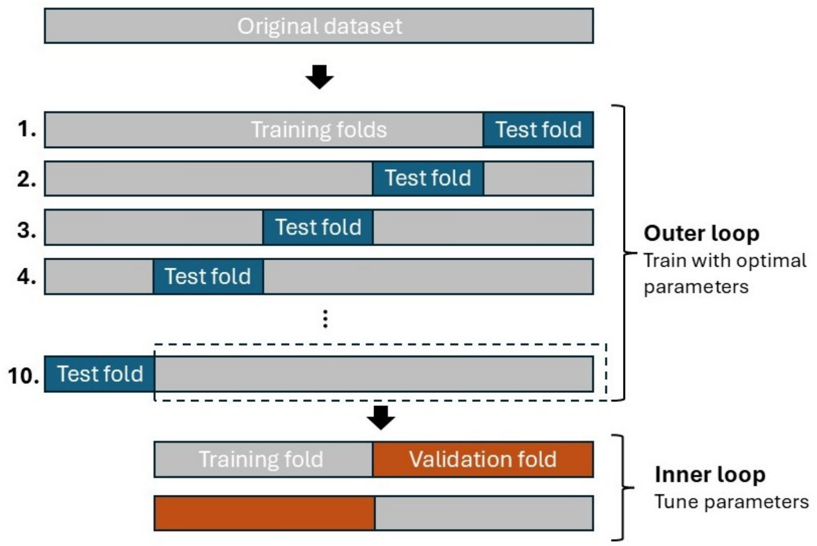

Figure S1: Schematic overview of 10-fold cross-validation strategy.

PCA scores plot of normalised human data, coloured for age

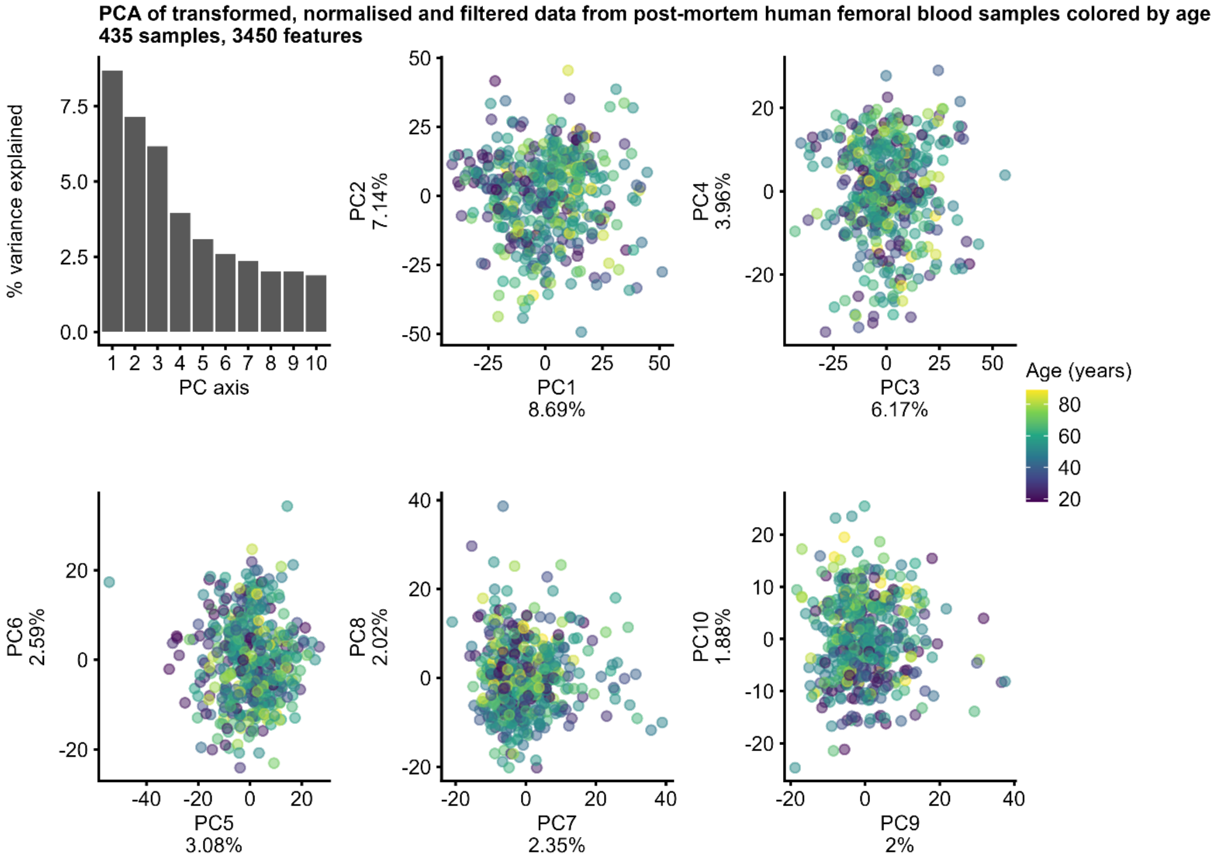

Figure S2: Scree plot and PCA scores plot of normalised human data showing the first 10 PC-axes coloured by age.

PCA scores plot of normalised human data, coloured for sex

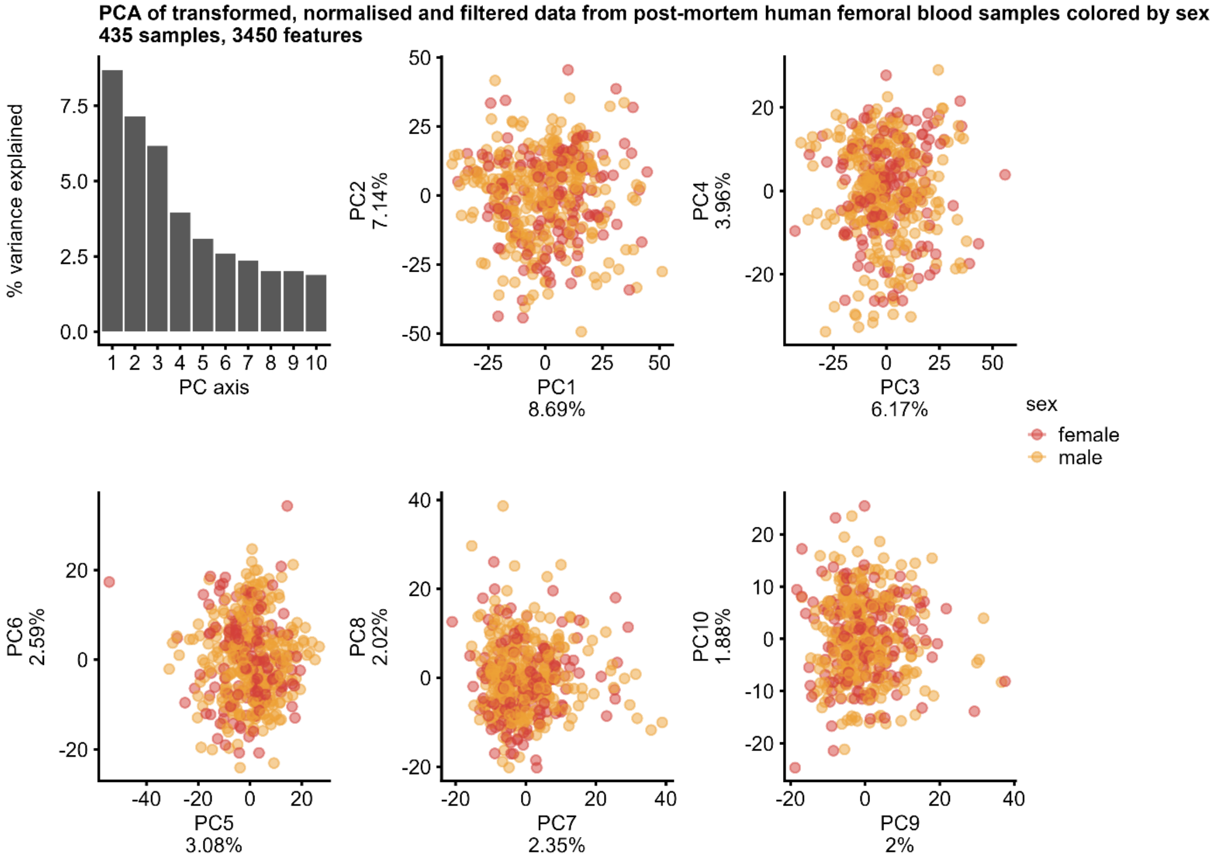

Figure S3: Scree plot and PCA scores plot of normalised human data showing the first 10 PC-axes coloured by sex.

PCA scores plot of normalised human data, coloured for body mass index (BMI)

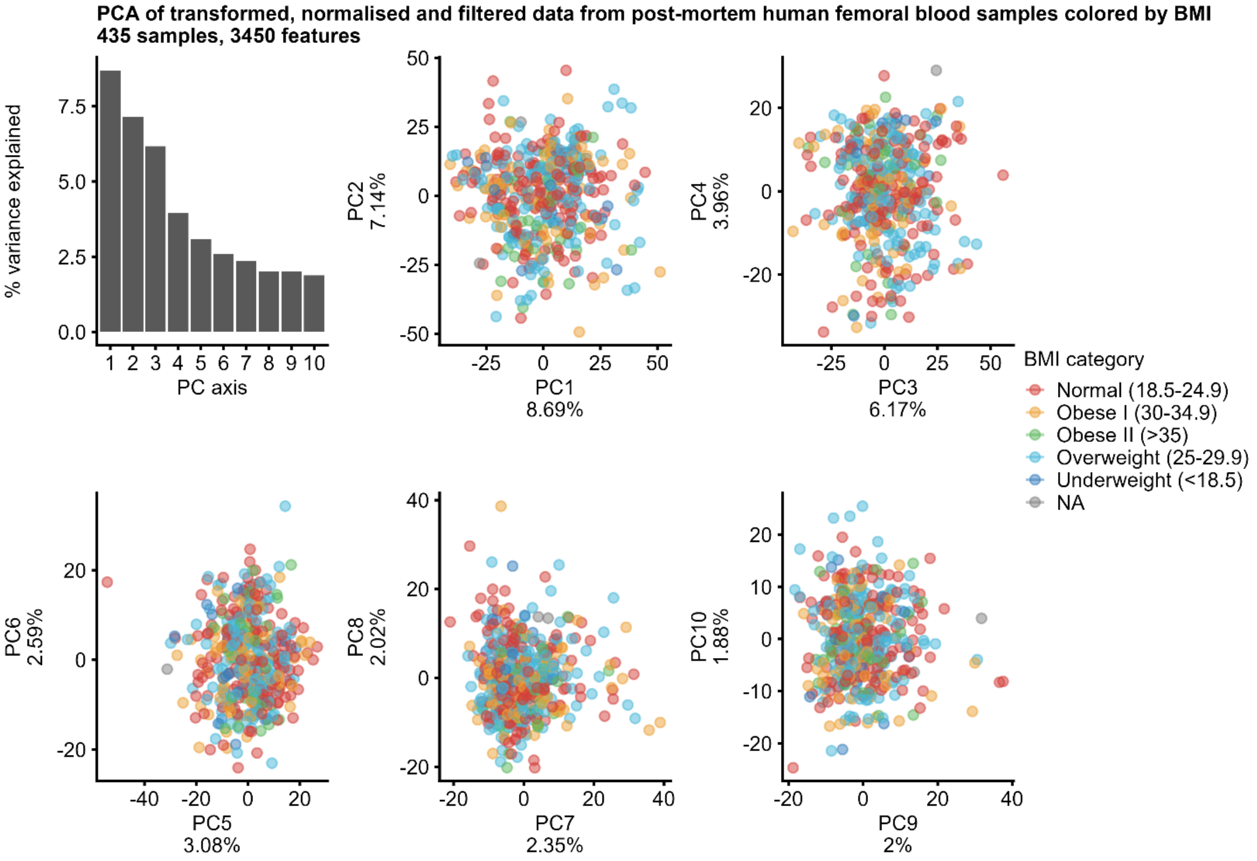

Figure S4: Scree plot and PCA scores plot of normalised human data showing the first 10 PC-axes coloured by body mass index (BMI) category.

Relative time-dependent changes in reanalysed rat data

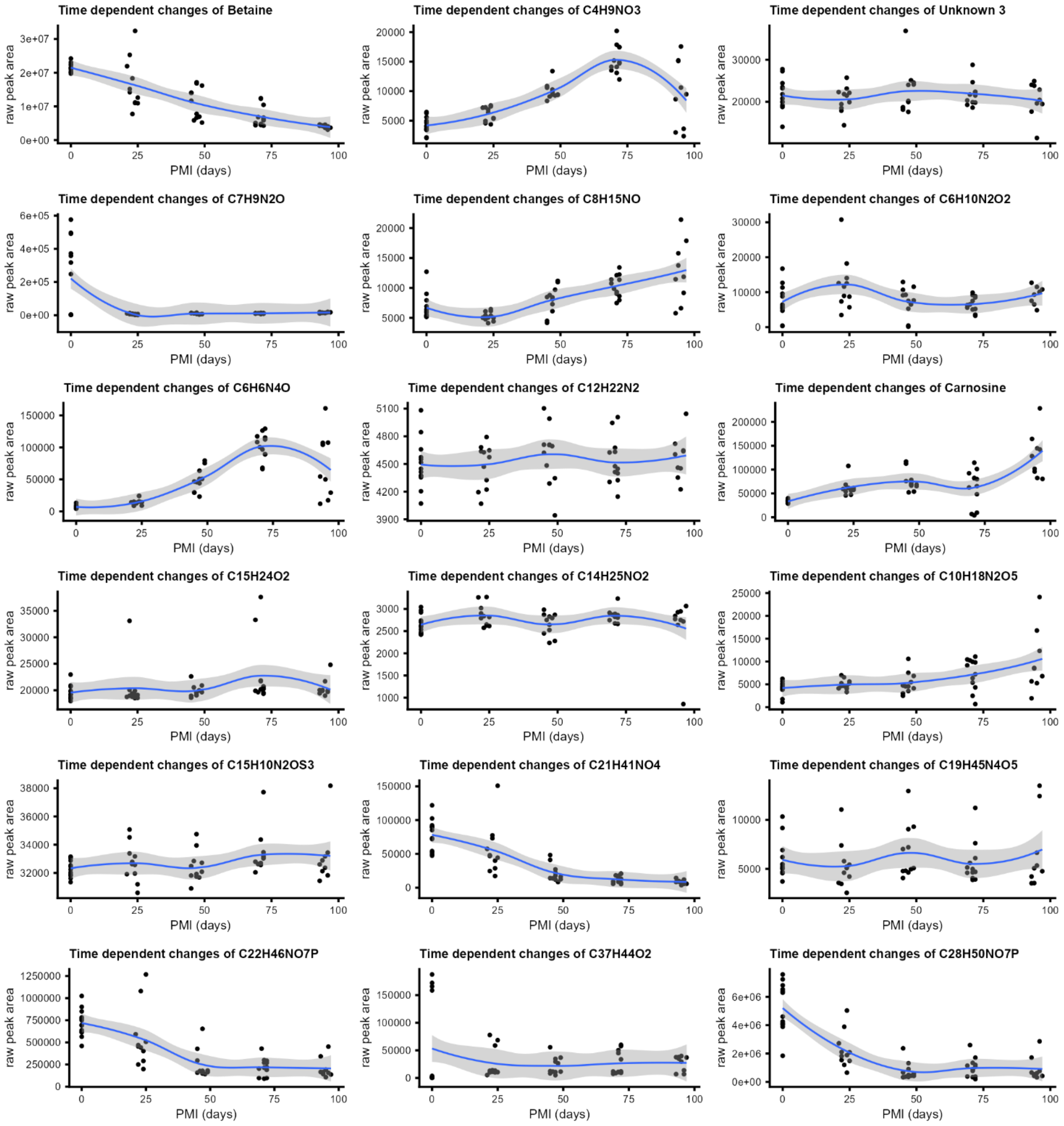

Figure S5: Relative time-dependent changes in rats of the 18 human-to-rat matched molecular features.

XCMS settings

prefiltering:
rtmin: 30 # Minimum retention time
rtmax: 660 # Minimum retention time
mzmin: 30 # Minimum Mz
mzmax: 1200 # Maximum Mz

centwave:
mzdiff: 0.01
peakwidth: c(3,30)
ppm: 30
prefilter: c(3,1000)
snthresh: 3

peak_grouping1:
binSize: 0.05
bw: 5
maxFeatures: 50
minFraction: 0.5

alignment:
extraPeaks: 5
family: gaussian
minFraction: 0.9
smooth: loess
span: 0.6

peak_grouping2:
binSize: 0.05
bw: 2.5
maxFeatures: 50
minFraction: 0.5
